# Therapeutic effects of mirodenafil, a phosphodiesterase 5 inhibitor, on stroke models in rats

**DOI:** 10.1016/j.neurot.2024.e00463

**Published:** 2024-10-11

**Authors:** Fred Kim, Padmanabh Singh, Hyunji Jo, Tianyang Xi, Dong-Keun Song, Sae Kwang Ku, Jai Jun Choung

**Affiliations:** a AriBio Co. Ltd., Seongnam-si 13535, Republic of Korea; b College of Korean Medicine, Daegu Haany University, Gyeongsan-si 38610, Republic of Korea

**Keywords:** Middle cerebral artery occlusion (MCAO), Mirodenafil, PDE5 inhibitor, Rats, Stroke

## Abstract

Mirodenafil is a phosphodiesterase 5 (PDE5) inhibitor with high specificity for its target and good blood-brain barrier permeability. The drug, which is currently used for treatment of erectile dysfunction, reduces Aβ and pTau levels and improves cognitive function in mouse models of Alzheimer's disease. In the present study, we investigated the effect of mirodenafil in the transient and permanent middle cerebral artery occlusion (tMCAO and pMCAO) models of stroke in rats. Starting 24 ​h after cerebral artery occlusion, mirodenafil was administered subcutaneously at doses of 0.5, 1, and 2 ​mg/kg per day for 9 days in the tMCAO model and for 28 days in the pMCAO model. Mirodenafil significantly increased sensorimotor and cognitive recovery of tMCAO and pMCAO rats compared to saline control rats, and significantly decreased the amount of degenerative cells and cleaved caspase-3 and cleaved PARP immunoreactive cells. Effects were seen in a dose-dependent manner up to 1 ​mg/kg mirodenafil. The benefits of mirodenafil treatment increased with longer treatment duration, and the largest improvements over control were typically observed on the last assessment day. There was no effect of mirodenafil on infarct volume in both tMCAO and pMCAO rats. In an experiment to determine the treatment window for mirodenafil effects, a protective effect was observed when treatment was delayed 72 ​h after MCAO, although the most improvement was observed with shorter treatment windows. Using pMCAO and tMCAO rat models of stroke, we determined that mirodenafil improves the recovery of sensorimotor and cognitive functions after MCAO and protects cortical cells from apoptosis and degeneration. Greater benefit was observed with longer duration of treatment, and improvement was seen even when treatment was delayed.

## Introduction

Stroke is the leading cause of serious disability in the world [1], impacting many millions of people. Ongoing efforts are aimed at ways to reduce brain damage and disability following stroke [2,3], although there remain few pharmacological treatments that are effective in stroke recovery.

Administration of a nitric oxide (NO) donor improves neurological functional recovery in rats after stroke via cGMP activation of CREB transcriptional signaling and other downstream mediators [[4], [5], [6]]. Phosphodiesterase 5 (PDE5) inhibitors function in a similar way to NO, by increasing the levels of cGMP and the activity downstream of protein kinase G [7].

PDE5 inhibitors have protective effects in the central nervous system through promotion of neurogenesis and synaptogenesis, and reduction of oxidative stress and neuroinflammation [8]. Potential therapeutic effects have been described for PDE5 inhibitors in Alzheimer's disease and other neurological conditions [9]. A single dose of the PDE5 inhibitor sildenafil increased cerebral blood flow and cerebral metabolic function in Alzheimer's disease patients [10].

The PDE5 inhibitor mirodenafil has good blood-brain barrier penetrability and high specificity for PDE5 [11]. Mirodenafil improved learning and memory, and reduced levels of Aβ42 and phosphorylated Tau in an Alzheimer's disease mouse model [12]. Studies in rodent models of stroke have found improved cerebral blood flow and cognitive performance following treatment with a PDE5 inhibitor [[13], [14], [15]]. In rat pups with hypoxia-ischemia, treatment with sildenafil for 7 days preserved the gross morphology of the hippocampus, protected mature neurons, and decreased apoptosis [16]. In the rat MCAO model of cerebral stroke, sildenafil treatment increased cGMP levels and improved neurogenesis and neurological functional recovery [17,18]. Other PDE5 inhibitors including tadalafil, yonkenafil, and PF-5 have also been found to improve recovery from stroke in rodent models [[19], [20], [21]].

In the present study, we examined the effect of mirodenafil on stroke recovery using rat middle cerebral artery occlusion (MCAO), the model of choice for approximating the pathology and symptoms of human stroke [22]. Our data suggest a potential therapeutic effect of mirodenafil for the treatment of stroke.

## Methods

### Animals and husbandry

Male Sprague-Dawley rats (6-wk old, 160–180 ​g upon receipt; SLC, Japan), were allocated 5 per polycarbonate cage and acclimatized for 16–100 days at 20–25 ​°C and 50–55 ​% humidity, with a 12 ​h:12 ​h light dark cycle. Standard rodent chow (Samyang, Korea) and water were supplied ad libitum. All animals were treated in accordance with the Guidelines for Care and Use of Laboratory Animals of Daegu Haany University based on the Guide for the Care and Use of Laboratory Animals by the Institute of Laboratory Animal Resources, Commission on Life Science, National Research Council, USA.

The study was divided into 3 parts: 1) dose-dependent effects of mirodenafil on transient MCAO (tMCAO); 2) dose-dependent effects of mirodenafil on permanent MCAO (pMCAO); and 3) time-dependent effects of mirodenafil on pMCAO.

Part 1 (10 animals per condition).

1. Sham-operated, administered saline (Sham control)
2. tMCAO-operated, administered saline (tMCAO control)
3. tMCAO-operated, administered 0.5 ​mg/kg mirodenafil
4. tMCAO-operated, administered 1.0 ​mg/kg mirodenafil
5. tMCAO-operated, administered 2.0 ​mg/kg mirodenafil

Part 2 (10 animals per condition).

1. Sham-operated, administered saline (Sham control)
2. pMCAO-operated, administered saline (pMCAO control)
3. pMCAO-operated, administered 0.5 ​mg/kg mirodenafil
4. pMCAO-operated, administered 1.0 ​mg/kg mirodenafil
5. pMCAO-operated, administered 2.0 ​mg/kg mirodenafil

Part 3 (10 animals per condition).

1. Sham-operated, administered saline (Sham control)
2. pMCAO-operated, administered saline starting 24 ​h later (pMCAO control)
3. pMCAO-operated, administered 1.0 ​mg/kg mirodenafil starting 24 ​h later
4. pMCAO-operated, administered 1.0 ​mg/kg mirodenafil starting 72 ​h later
5. pMCAO-operated, administered 1.0 ​mg/kg mirodenafil starting 168 ​h later

### Preparations and administration of mirodenafil

Mirodenafil 2HCl (SK Chemical Life Science Business, Korea) was dissolved in saline at up to 2 ​mg/ml and stored at 4 ​°C protected from light and humidity. For the two dose-dependent studies (Parts 1 and 2), 0.5, 1, and 2 ​mg/kg of mirodenafil were dissolved in saline and subcutaneously (s.c.) administered 24 ​h after surgery in a volume of 1 ​ml/kg on the dorsal back skin, once a day for 9 days (tMCAO) or once a day for 28 days (pMCAO). For the time-dependent study, mirodenafil (1 ​mg/kg, s.c.) was administered from 24, 72, and 168 ​h after pMCAO, once a day for 28 days. In saline control rats, equal volumes of saline as vehicle were administered.

### MCAO

The procedures for tMCAO and pMCAO were performed according to published methods [23,24]. All animals were anesthetized with 2–3 ​% isoflurane (Hana Pharm. Co., Hwasung, Korea) in 70 ​% N_2_O and 28.5 ​% O_2_, and were maintained with 1–1.5 ​% isoflurane in a mixture of 70 ​% N_2_O and 28.5 ​% O_2_. Under anesthesia, the right common carotid artery, external carotid artery (ECA), and internal carotid artery (ICA) were exposed and separated from the vagus nerve. A silicon rubber-coated monofilament (4–0 fine MCAO suture L56 PK10; Doccol, Redlands, CA, USA) was inserted into the ICA via ECA to occlude the middle cerebral artery (MCA) located 23–24 ​mm from the bifurcation of ICA and ECA. At 60 ​min after MCAO, blood reperfusion was achieved by withdrawing the suture from ECA. Core body temperature was maintained at 37.0 ​± ​0.5 ​°C using a heating blanket (Harvard, Holliston, MA, USA) and a heat lamp during and for 2 ​h after surgery. Mortality was less than 5 ​%. For sham tMCAO, all procedures were performed in the same way, with the exception of the occlusion and reperfusion of the MCA using monofilament. For pMCAO surgery, enrofloxacin (5 ​mg/kg; Neotril-50 injection, SamU Median Co., Yesan, Korea) was given intraperitoneally before the surgery to prevent infections. After that the temporalis muscle was bisected and reflected through an incision made midway between the eye and the eardrum canal. The proximal MCA was exposed through a subtemporal craniectomy by microdental drills (Saeshin, Daegu, Korea) without removing the zygomatic arch and without transecting the facial nerve. The artery was then occluded by microbipolar coagulation from just proximal to the olfactory tract to the inferior cerebral vein, and was transected. Mortality was less than 5 ​%. For sham pMCAO, all procedures were performed in the same way, with the exception of the occlusion of the MCA.

### Assessment of sensorimotor function

The investigator performing the surgery and behavioral assessments was blinded to treatment assignment. The effects of tMCAO and pMCAO on sensorimotor function were evaluated using limb placing [25] and body swing [26] tests. These tests were performed one day before surgery to obtain a baseline measurement, and then on Days 1, 3, 7 and 10 after tMCAO or Days 1, 3, 7, 14, 21, and 28 after pMCAO. Behavioral tests were done before drug administration on days when both were scheduled.

Limb placing test: Forelimb and hindlimb placement was assessed independently. For the forelimb placing test, the rats were held by their torso with their forepaws hanging free and moved slowly toward the edge of a tabletop, stopping short of touching the vibrissae (for vision-induced placing), touching the vibrissae (for vibrissae-induced placing), making light contact with the front of the forepaw to the edge of the tabletop (for tactile-induced placing), and pressing the forepaws to the edge of the table with increased pressure (for proprioceptive-induced placing). Hindlimb placing was conducted in the same manner as above but with tactile and proprioceptive stimulation applied to the front/top of each hindlimb. Each limb placement in response to visual, vibrissae, tactile, and proprioceptive stimulation was scored in the following manner: 0 ​= ​normal performance, 1 ​= ​performance with unilateral limb, 2 ​= ​performance with a delay (2 ​s) and/or incomplete, 3 ​= ​no performance. A total of 12 points in forelimb placing test and 6 points in hindlimb placing test corresponded to maximal neurological deficit, and 0 points corresponded to normal performance.

Body swing tests: Animals were held approximately 2 ​cm from the base of the tail and elevated to an inch above a surface of a table. A swing was recorded whenever the rat moved its head out of the vertical axis to either side by more than 10° from vertical and then returned to the vertical position. Thirty total swings were counted per animal. tMCAO was administered on the right hemisphere, which tends to cause animals to swing to the contralateral (left) side. The percentage of body swings to the ipsilateral (right) side was recorded as a measure of recovery, with a functionally normal animal scoring approximately 50 ​%.

### Assessment of cognitive motor behavior

Cognitive testing was conducted using the water maze task [27]. On Day 10 after tMCAO and on Days 14 and 28 after pMCAO, rats were administered 3 trials, 10 ​min apart, in a large dark-colored tank (150 ​cm in diameter ​× ​50 ​cm in height) filled with clear water at a temperature of 22.0 ​°C ​± ​1.0 ​°C. A 15 ​× ​30 ​cm submerged platform (2 ​mm below water surface) was placed in the northwest quadrant of the pool. The release point was always the southern end of the pool. The rats were lowered into the pool facing the wall and were released. The swim paths of the rats for each trial were recorded with a computer interfaced camera tracking system (Smart Junior, PanLab, Barcelona, Spain), and the distance (m) and time (sec) taken to reach the escape platform were measured.

### Histopathology

On Day 10 after tMCAO and Day 29 after pMCAO, brains dissected into 6 coronal sections (2 ​mm thickness) ranging from 2 to 14 ​mm from the frontal brain pole on the rat brain stainless steel coronal matrix (Harvard, MA, USA) were directly fixed in 10 ​% neutral buffered formalin (NBF), not stained with triphenyl tetrazolium chloride (TTC), embedded in paraffin, cross-sectioned, and stained with hematoxylin and eosin (H&E) for general histopathology of cerebral cortex. Under H&E stain, the brain atrophic % and the numbers of degenerative neurons (as seen as eosinophilic) were calculated by histomorphometry. The histopathologist performing the analysis was blinded to treatment assignments.

Histomorphometry: The atrophic % of ipsilateral cerebral cortex was calculated and compared to intact contralateral hemisphere in prepared peri-infarct histological specimens using a computer-assisted image analysis program. In addition, the numbers of degenerative neurons were measured in the restricted view fields of cerebral cortex. Cerebral atrophy formation = (contralateral cerebral cortex area − ipsilateral cerebral cortex area)/contralateral cerebral cortex area ​× ​100 (%)

### Immunohistochemistry

After de-paraffinization of prepared peri-infarct/defect regions of cerebral histological paraffin sections, antigen (epitope) retrieval was performed using 10 ​mM citrate buffer (pH 6.0) at 95∼100 ​°C. Endogenous peroxidase activity was blocked (methanol and 0.3 ​% H_2_O_2_ for 30 ​min), sections were rinsed 3 ​× ​in 0.01 ​M PBS pH 7.2 and incubated with normal horse serum blocking solution (Vector Lab. Inc., Burlingame, CA, USA) at 1:1000 dilution for 1 ​h followed by incubation with primary antisera (anti-cleaved caspase-3 [Asp175] (1:400) or ant-cleaved PARP [Asp214] (1:100); Cell Signaling Technology, Boston, MA USA) overnight at 4 ​°C in a humidity chamber. After, sections were rinsed 3 ​× ​in 0.01 ​M PBS and incubated with biotinylated universal secondary antibody (Vector Lab) (1:50) for 1 ​h at RT in a humidity chamber followed by 3 ​× ​rinses in 0.01 ​M PBS. Sections were incubated in ABC reagents (Vectastain Elite ABC Kit, Vector Lab) at 1:50 for 1 ​h at RT in a humidity chamber, rinsed 3 ​× ​in 0.01 ​M PBS and incubated in peroxidase substrate kit (Vector Lab) for 5 ​min at RT, rinsed 3 ​× ​in 0.01 ​M PBS and counterstained with Mayer's hematoxylin solution. The sections were then rinsed in running tap water for 30 ​min, dehydrated with 95 ​% ethanol for 2 ​min, 100 ​% ethanol 3 times, cleared in xylene twice and processed with coverslips secured with permanent mounting medium and observed under light microscope (Nikon, Tokyo, Japan).

Histomorphometry: The nerve cells occupied by over 10 ​% of immunoreactivities of each antiserum, caspase-3 and PARP were regarded as immune-reactive. The numbers of caspase-3 and PARP-immuno-reactive cells per mm^2^ of ipsilateral peri-infarct cerebral cortex were measured using a computer-assisted image analysis program.

### Statistical analyses

All data were expressed as mean ​± ​standard error of mean (SEM) of 10 rats. Data were analyzed and graphs plotted by comparing sham control with respect to tMCAO/pMCAO control and tMCAO/pMCAO control with respect to dose groups. Two-way analysis of variance (ANOVA) with multiple comparison tests for different dose groups were conducted using Tukey's post-hoc test. Statistical analyses were conducted using PRISM statistical software for Windows.

## Results

### Sensorimotor recovery following MCAO

**Fig. 1:**
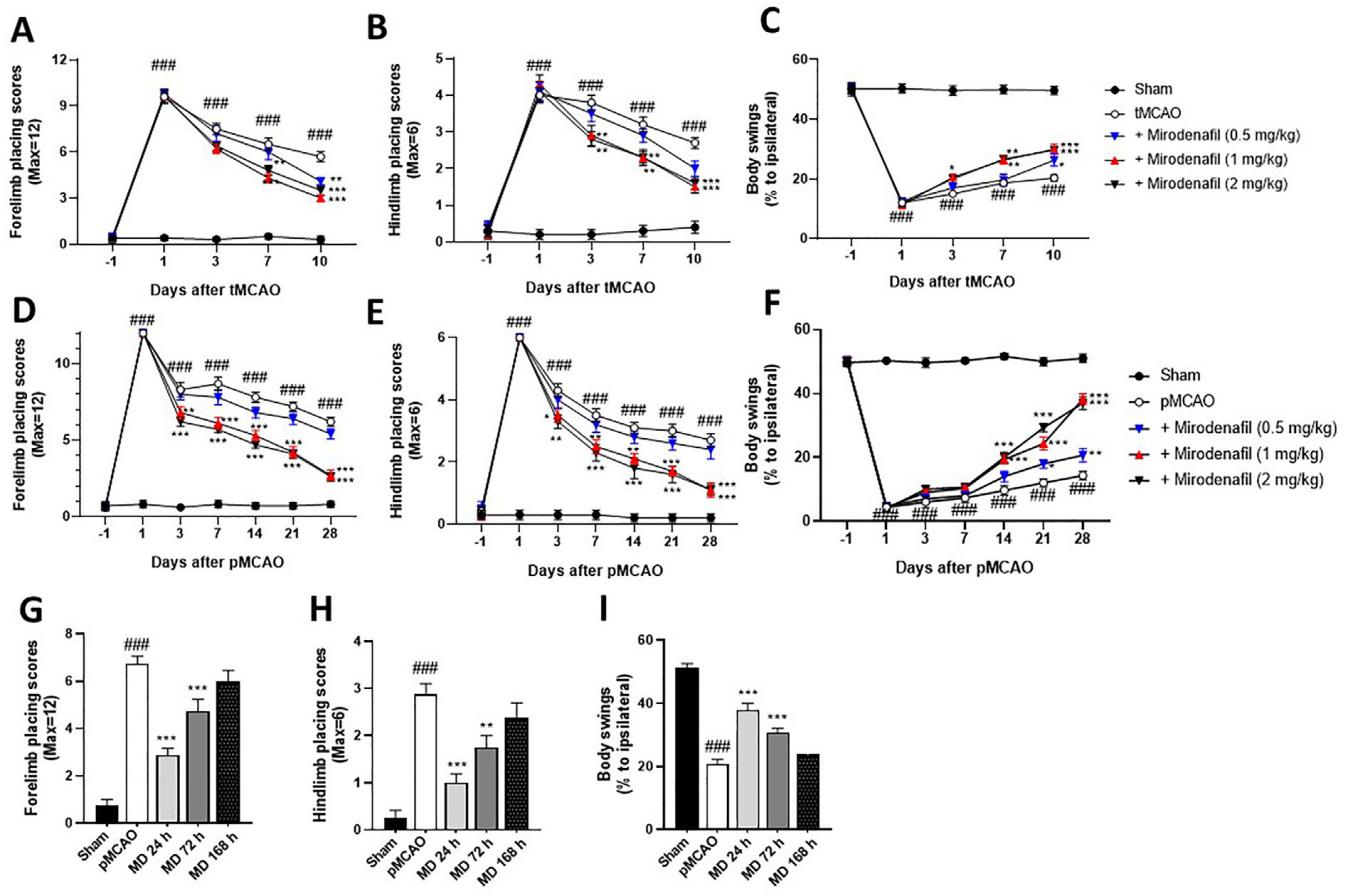
Mirodenafil-induced improvement in sensorimotor functions in tMCAO (A–C) and pMCAO (D–I) rats. Forelimb placing test (A,D,G), hindlimb placing test (B,E,H), body swings to the ipsilateral side (C,F,I). Different doses of mirodenafil was subcutaneously (s.c.) administered 24 ​h after surgery once a day for 9 days (A–C) or for 28 days (D–F). For the therapeutic time window experiments, mirodenafil (1 ​mg/kg, s.c.) was administered from 24, 72, and 168 ​h after pMCAO for 28 days (G–I). The data are expressed as mean ​± ​s.e.m. with n ​= ​10 mice per group. ###p ​< ​0.001 compared to sham control; ∗p ​< ​0.05, ∗∗p ​< ​0.01 and ∗∗∗p ​< ​0.001 compared to tMCAO or pMCAO control.

**Fig. 2:**
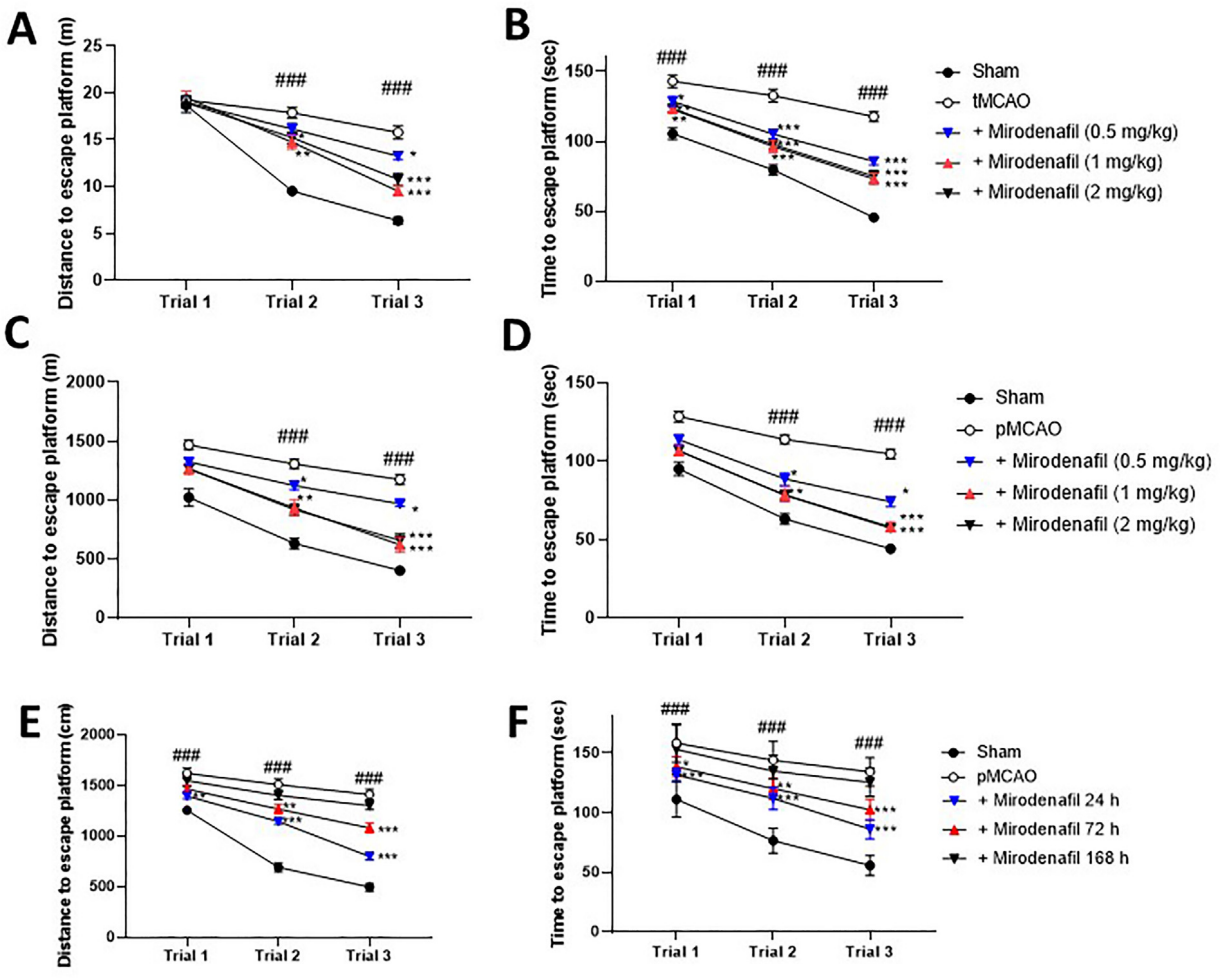
Mirodenafil-induced improvement in cognitive functions in water maze test in tMCAO (A,B) and pMCAO (C–F) rats. Different doses of mirodenafil was subcutaneously (s.c.) administered 24 ​h after surgery once a day for 9 days (A,B) or for 28 days (C,D). For the therapeutic time window experiments, mirodenafil (1 ​mg/kg, s.c.) was administered from 24, 72, and 168 ​h after pMCAO for 28 days (E,F). Three replicates of the water maze test were performed on day 10 (A,B) and day 28 (C–F) post-injury. The data are expressed as mean ​± ​s.e.m. with n ​= ​10 mice per group. ###p ​< ​0.001 compared to sham control; ∗p ​< ​0.05, ∗∗p ​< ​0.01 and ∗∗∗p ​< ​0.001 compared to tMCAO or pMCAO control.

tMCAO control rats showed sensorimotor deficits compared to sham controls as evident from increased forelimb and hindlimb placing scores and decreased percentage of body swings to the ipsilateral right sides (Fig. 1A–C). As shown in Fig. 1, Fig. 2 mg/kg mirodenafil significantly alleviated the tMCAO-induced increase in the forelimb placement scores from Day 7. In the hindlimb placing test, 1 and 2 ​mg/kg mirodenafil had significant effects from Day 3 (Fig. 1B). Similarly, in the body swing test, 1 and 2 ​mg/kg mirodenafil significantly alleviated the tMCAO-induced decrease in ipsilateral swings from Day 3, whereas 0.5 ​mg/kg mirodenafil had significant effects at Day 10 (Fig. 1C). In all tests, a similar degree of therapeutic effect was observed at 1 and 2 ​mg/kg mirodenafil.

In the pMCAO model, mirodenafil at 1 and 2 ​mg/kg significantly alleviated the increase in forelimb and hindlimb placing scores starting on Day 3 (Fig. 1D and E). In the body swing test, 1 and 2 ​mg/kg mirodenafil significantly alleviated the pMCAO-induced decrease from Day 14, with both doses having nearly the same degree of protective effect, while mirodenafil at 0.5 ​mg/kg significantly alleviated the pMCAO-induced decrease in the body swing test from Day 21 (Fig. 1F). At Day 28, animals treated with 1 ​mg/kg mirodenafil had recovered to 38 ​% ipsilateral swings, compared to 14 ​% for saline-treated control animals (Fig. 1F).

In the therapeutic time window experiments, when rats were examined on Day 28 after pMCAO, 1 ​mg/kg mirodenafil treatment significantly alleviated the sensorimotor deficits in forelimb and hindlimb placing tests (Fig. 1G and H) and body swings to ipsilateral side (Fig. 1I) when administered 24 ​h or 72 ​h but not 168 ​h after pMCAO.

### Cognitive recovery following MCAO

We evaluated the effects of mirodenafil on the MCAO-induced impairment of cognitive motor behaviors using water maze test 10 days (tMCAO) or 28 days (pMCAO) post-injury. In the tMCAO model, all 3 doses of mirodenafil significantly alleviated impairment in both distance traveled and time to reach the escape platform (Fig. 2A and B). As with sensorimotor functions (Fig. 1), the beneficial effects of mirodenafil were dose-dependent up to 1 ​mg/kg and comparable at 1 and 2 ​mg/kg. The same pattern was observed in the pMCAO model (Fig. 2C and D).

In the therapeutic window experiments, there was significant recovery of performance in the water maze test on Day 28 in pMCAO rats treated with 1 ​mg/kg mirodenafil 24 ​h and 72 ​h but not 168 ​h after surgery (Fig. 2E and F).

### Body weights

Mirodenafil 1 and 2 ​mg/kg significantly enhanced the recovery from the surgery-induced decrease in body weights in both tMCAO and pMCAO rats (Supplemental Fig. 1A and B). For the therapeutic window experiment, mirodenafil (1 ​mg/kg) 24 ​h, 72 ​h, but not 168 ​h after insult showed a significantly enhanced recovery from the surgery-induced decrease in body weights (Supplemental Fig. 1C).

### Infarct volume

We examined the effects of mirodenafil on the MCAO-induced focal infarct volume in the cerebral cortex on Day 10 post-injury for tMCAO and on Day 29 post-injury for pMCAO, and found no significant change on the infarct volume in mirodenafil-treated rats compared to tMCAO or pMCAO control rats (Supplemental Fig. 2).

### Peri-infarct cerebral cortex histopathology

**Fig. 3:**
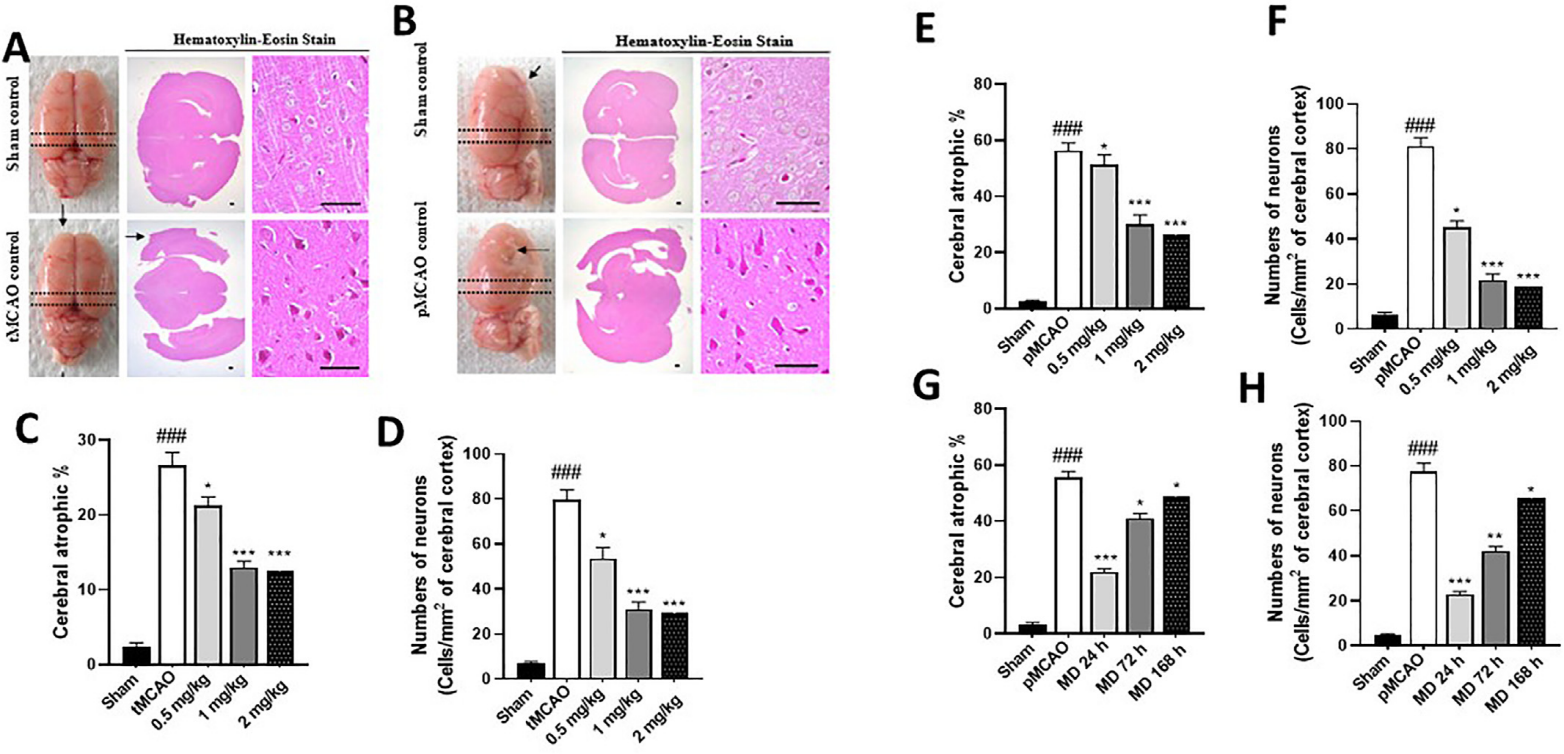
Mirodenafil-induced decrease in cerebral atrophic % and degenerative cells. Representative histology in tMCAO (A) and pMCAO (B) rats. Scale bars denote 100 ​μm. Cerebral atrophic % (C,E,G) and numbers of degenerative cells (D,F,H) in brain sections of sham, tMCAO rats on day 10 post-injury (C,D) and pMCAO rats on day 29 post-injury (E–H). Different doses of mirodenafil was subcutaneously (s.c.) administered 24 ​h after surgery once a day for 9 days (C,D) or for 28 days (E,F). For the therapeutic time window experiments, mirodenafil (1 ​mg/kg, s.c.) was administered from 24, 72, and 168 ​h after pMCAO for 28 days (G,H). The data are expressed as mean ​± ​s.e.m. with n ​= ​10 mice per group. ###p ​< ​0.001 compared to sham control; ∗p ​< ​0.05 and ∗∗∗p ​< ​0.001 compared to tMCAO or pMCAO control.

We examined the effects of mirodenafil on peri-infarct cerebral cortex histopathology after MCAO. Both tMCAO (Fig. 3A) and pMCAO (Fig. 3B) surgeries resulted in increased atrophy in the cortex and increased degenerative cells, as determined by eosinophilic staining, relative to sham-treated animals. More severe cerebral damage was observed in the pMCAO model.

All doses of mirodenafil significantly reduced MCAO-related cerebral atrophy and degenerative cells when examined on Day 10 after tMCAO (Fig. 3C) and Day 28 after pMCAO (Fig. 3D). In the tMCAO model, there was a 2.1 ​× ​decrease (12 ​% vs. 26 ​%) in cerebral atrophy, and a 2.6 ​× ​decrease (31 vs. 79 ​cells/mm^2^) in degenerative cells in 1 ​mg/kg mirodenafil-treated animals compared to tMCAO control animals.

The results were similar in pMCAO rats. There was a 1.9 ​× ​reduction in cerebral atrophy (30 ​% vs. 56 ​%), and a 3.8 ​× ​reduction (21 vs. 81 ​cells/mm^2^) in the number of degenerative cells in rats treated with 1 ​mg/kg mirodenafil compared to controls rats. As with the sensorimotor and cognitive experiments, there was a dose-dependent effect up to 1 ​mg/kg and comparable effects at 1 and 2 ​mg/kg.

When the therapeutic window of treatment was examined, there was a time-dependent effect of 1 ​mg/kg mirodenafil administration in limiting cortical atrophy and degenerative cells. The largest recovery was observed when treatment was observed 24 ​h after MCAO, but significant reductions were also observed when treatment was administered after 72 ​h or 168 ​h.

### Apoptotic cell markers

**Fig. 4:**
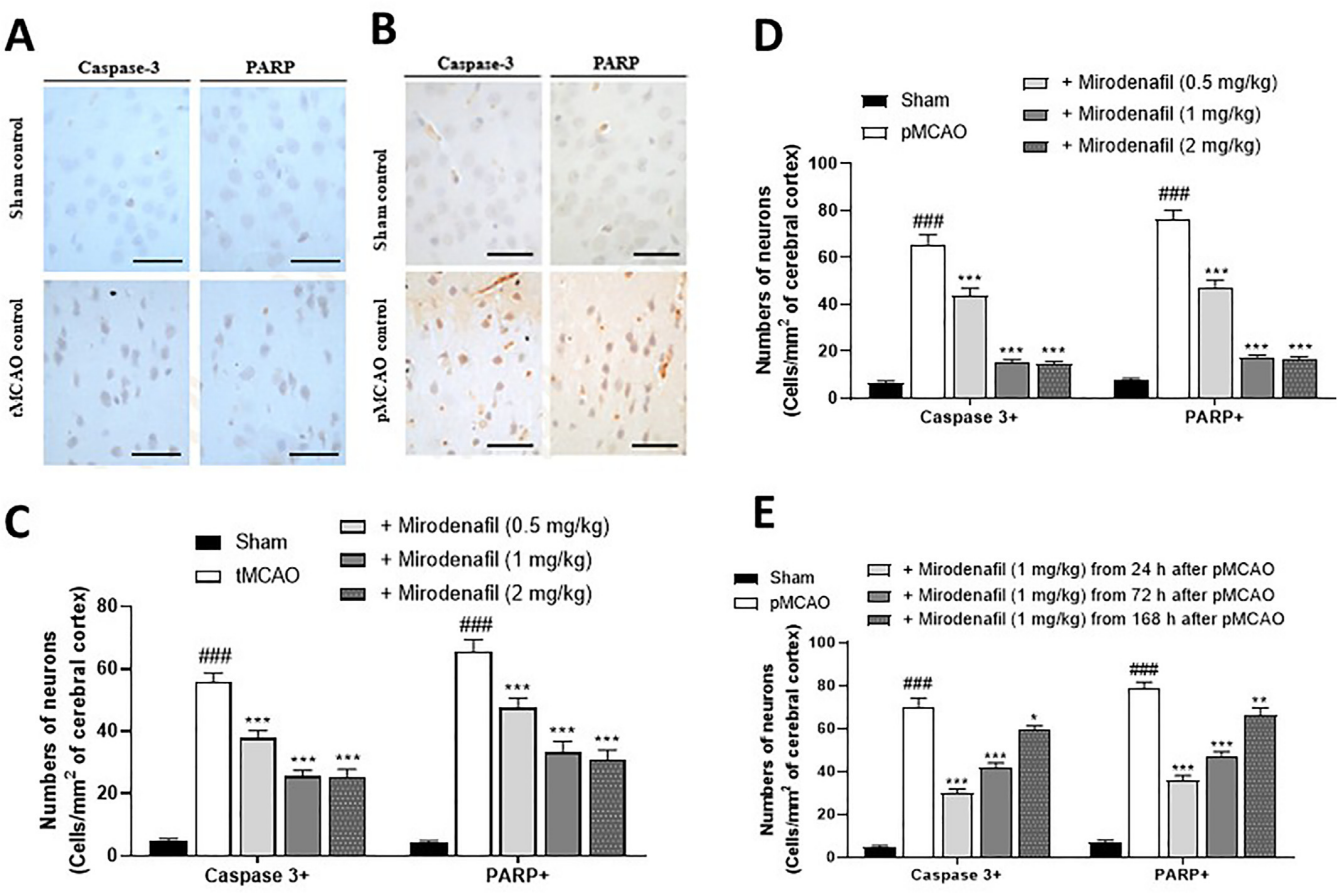
Mirodenafil-induced decrease in caspase-3 and cleaved PARP-immunoreactive neurons. Representative histology in tMCAO (A) and pMCAO (B) rats. Scale bars denote 100 ​μm. Caspase-3 and cleaved PARP immunoreactive neurons in brain sections of sham, tMCAO rats on day 10 post-injury (A,C) and pMCAO rats on day 29 post-injury (B,D,E). Different doses of mirodenafil was subcutaneously (s.c.) administered 24 ​h after surgery once a day for 9 days (C) or for 28 days (D). For the therapeutic time window experiments, mirodenafil (1 ​mg/kg, s.c.) was administered from 24, 72, and 168 ​h after pMCAO for 28 days (E). The data are expressed as mean ​± ​s.e.m. with n ​= ​10 mice per group. ###p ​< ​0.001 compared to sham control; ∗p ​< ​0.05, ∗∗p ​< ​0.01, and ∗∗∗p ​< ​0.001 compared to tMCAO or pMCAO control.

We asked if treatment with mirodenafil protected cortical cells from apoptosis using antibody staining for caspase-3 and cleaved PARP. Quantitative assessment found >10 ​× ​increase in immunoreactivity in tMCAO (Fig. 4A) and pMCAO (Fig. 4B) animals compared to sham-treated animals. Treatment with mirodenafil significantly reduced the number of caspase-3 and cleaved PARP immunoreactive cells in tMCAO and pMCAO rats in a dose-dependent manner (Fig. 4C and D).

When the therapeutic window of treatment was examined, there was a significant decrease in immunoreactivity at all treatment times, though greater recovery was achieved with more rapid treatment, as seen in other experiments (Fig. 4E).

## Discussion

Given the marked impact that stroke has on people's well-being, there is urgent interest in developing therapeutic agents that minimize the damage from stroke and enhance recovery. The present study found beneficial effects of mirodenafil, a PDE5 inhibitor, in measures of sensorimotor, cognitive, and histopathological recovery following stroke in both tMCAO and pMCAO models of stroke in rats. We consistently found a dose-dependent effect of mirodenafil up to 1 ​mg/kg, with comparable effects at 1 ​mg/kg and 2 ​mg/kg. Therefore the dose of 1 ​mg/kg mirodenafil is considered to be sufficiently effective.

The effects of mirodenafil on recovery of sensorimotor performance (Fig. 1) appear to be rapid, as significant differences from control animals in forelimb and hindlimb placing tests were observable by Day 3 when treatment was initiated 24 ​h after surgery. Generally, a greater benefit of mirodenafil was observed with longer post-MCAO treatment, and the largest improvement over control animals was typically observed at the last assessment (Day 10 for tMCAO, Day 28 for pMCAO). We used water maze test to assess cognitive recovery 10 days after tMCAO and 14 and 28 days after pMCAO. In both time and distance to reach the escape platform, we again found significant improvement over control animals following administration of 1 ​mg/kg and 2 ​mg/kg mirodenafil. When the window of treatment was examined, there was the expected finding that treatment 24 ​h after surgery produced the most rapid and extensive recovery of sensorimotor and cognitive defects, although significant benefit was also found when treatment was administered 72 ​h after surgery.

Histopathological assessments were conducted to determine the extent of brain damage following MCAO. Using cortical slices and cellular staining, we found reduction in cortical atrophy and degenerative cells following administration of 1 ​mg/kg and 2 ​mg/kg mirodenafil compared to saline treatment. Supporting the histopathological findings, we found that cellular apoptosis in MCAO rats, as measured by cleaved caspase-3 and cleaved PARP immunohistochemistry, was reduced by treatment with mirodenafil. In evaluating the time window of treatment, the greatest improvement was observed when animals were treated with 1 ​mg/kg mirodenafil 24 ​h after MCAO, though improvement over control animals was also observed when mirodenafil was administered 72 ​h or 168 ​h after MCAO.

There was no effect of mirodenafil on infarct volume, which is consistent with previous experiments with sildenafil or tadalafil that also showed no effect when administered 24 ​h after pMCAO [17,20]. However, when a single dose of sildenafil was administered intraperitoneally 5 ​min after pMCAO, a significant decrease in tissue loss was observed on Day 8[28], and a single dose of sildenafil injected intraperitoneally 30 ​min after reperfusion of MCAO surgery effectively reduced infarct volume on Day 3 post-injury [29]. Inhibition of infarction was also observed when yonkenafil, a PDE5 inhibitor, was administered 4 ​h after stroke [21]. Thus, it is possible that mirodenafil administration started earlier than 24 ​h after stroke may also reduce infarct volume.

Our data are consistent with other studies that have found PDE5 inhibitors increase recovery from stroke [[13], [14], [15]]. PDE5 acts via the cGMP pathway to increase CREB transcriptional activity. The effects that have been attributed to PDE5 inhibitors for stroke recovery are increased synaptogenesis [5], neurogenesis [17,20,30], angiogenesis [5,20,31], improved cerebral blood flow [31], and reduced apoptosis [21,32]. We previously found that mirodenafil activates WNT signaling in neurons [12] and inhibits pro-inflammatory cytokine production in microglia (unpublished data), both of which also factor into cerebral ischemia/reperfusion injury [33,34].

In conclusion, using pMCAO and tMCAO rat models of stroke, we found that mirodenafil improves the recovery of sensorimotor and cognitive functions after MCAO and protects cortical cells from apoptosis and degeneration. Further research is planned to assess the effects of mirodenafil of treatment of stroke.

## Author contributions

JJC conceived and SKK did the experiments. FK, PS, and HJ analyzed the data. TX, and DKS wrote the manuscript. The final manuscript was approved by all authors. We thank A. Schindler for critical review of the manuscript.

## Funding

This research was supported by a grant from the Korea Health Technology R&D Project through the Korea Health Industry Development Institute (KHIDI), funded by the Ministry of Health & Welfare, Republic of Korea (Grant Number: HR22C141104).

## Declaration of competing interest

The authors declare no competing interests.
