# Supplementary material for: Therapeutic effects of mirodenafil, a phosphodiesterase 5 inhibitor, on stroke models in rats
Source: Neurotherapeutics. 2024 Oct 11;22(1):e00463. doi: 10.1016/j.neurot.2024.e00463 (PMC11742844; doi:10.1016/j.neurot.2024.e00463)
Supplement: Multimedia component 1 [file mmc1.pptx]

## Slide 1
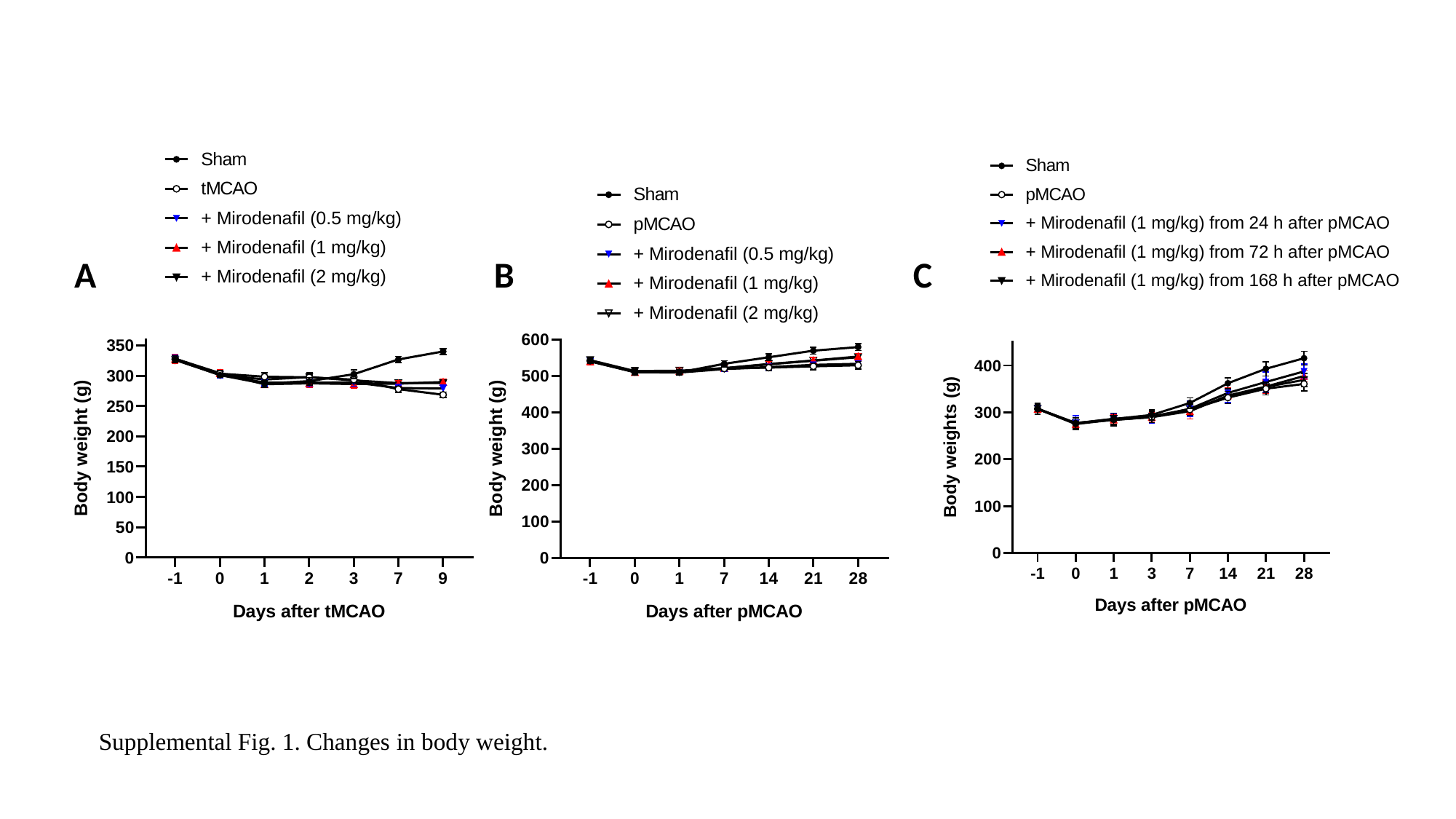

A
B
C
Supplemental Fig. 1. Changes in body weight.

## Slide 2
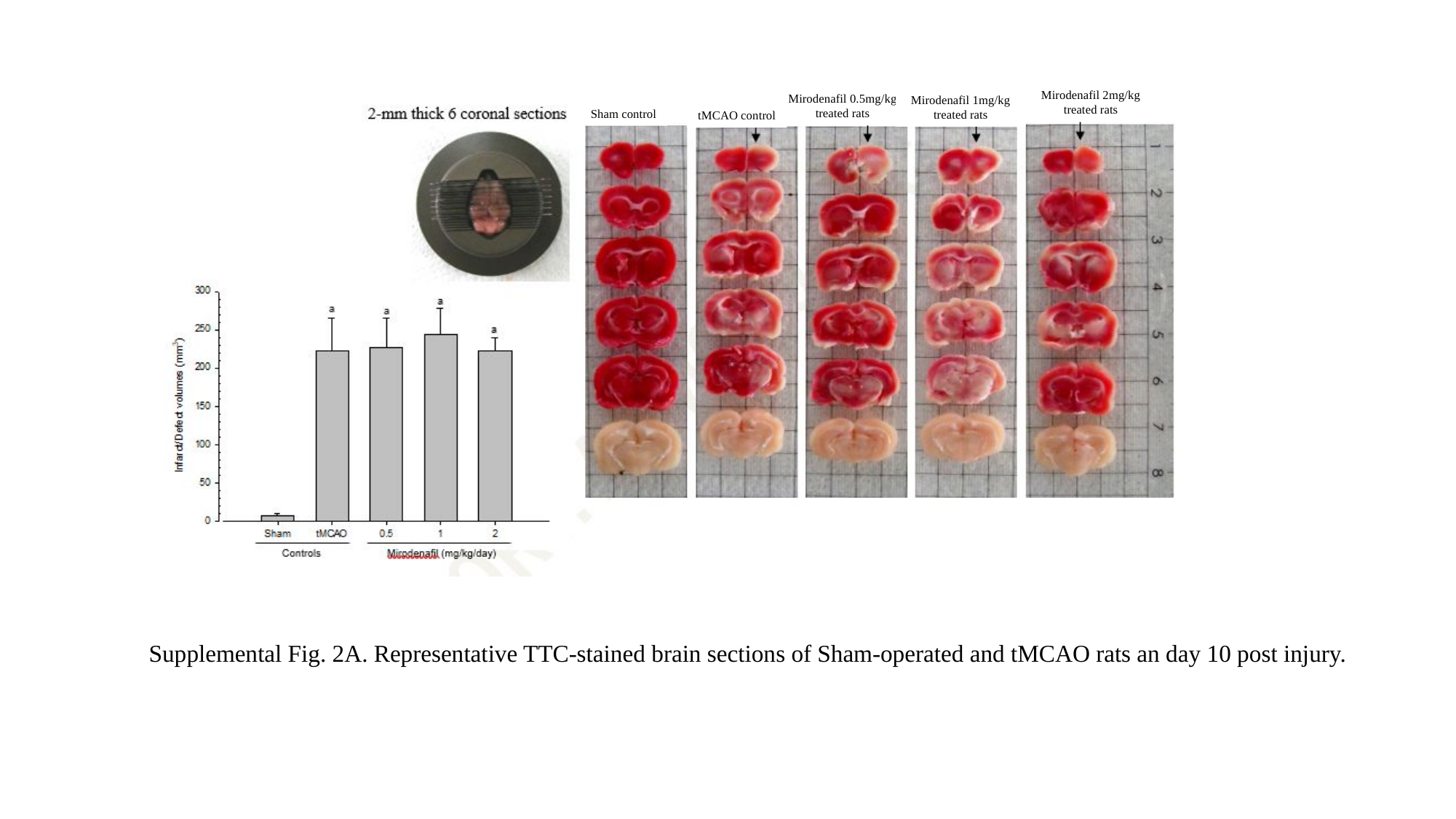

Mirodenafil 2mg/kg
treated rats
Mirodenafil 0.5mg/kg
treated rats
Mirodenafil 1mg/kg
treated rats
Sham control
tMCAO control
Supplemental Fig. 2A. Representative TTC-stained brain sections of Sham-operated and tMCAO rats an day 10 post injury.

## Slide 3
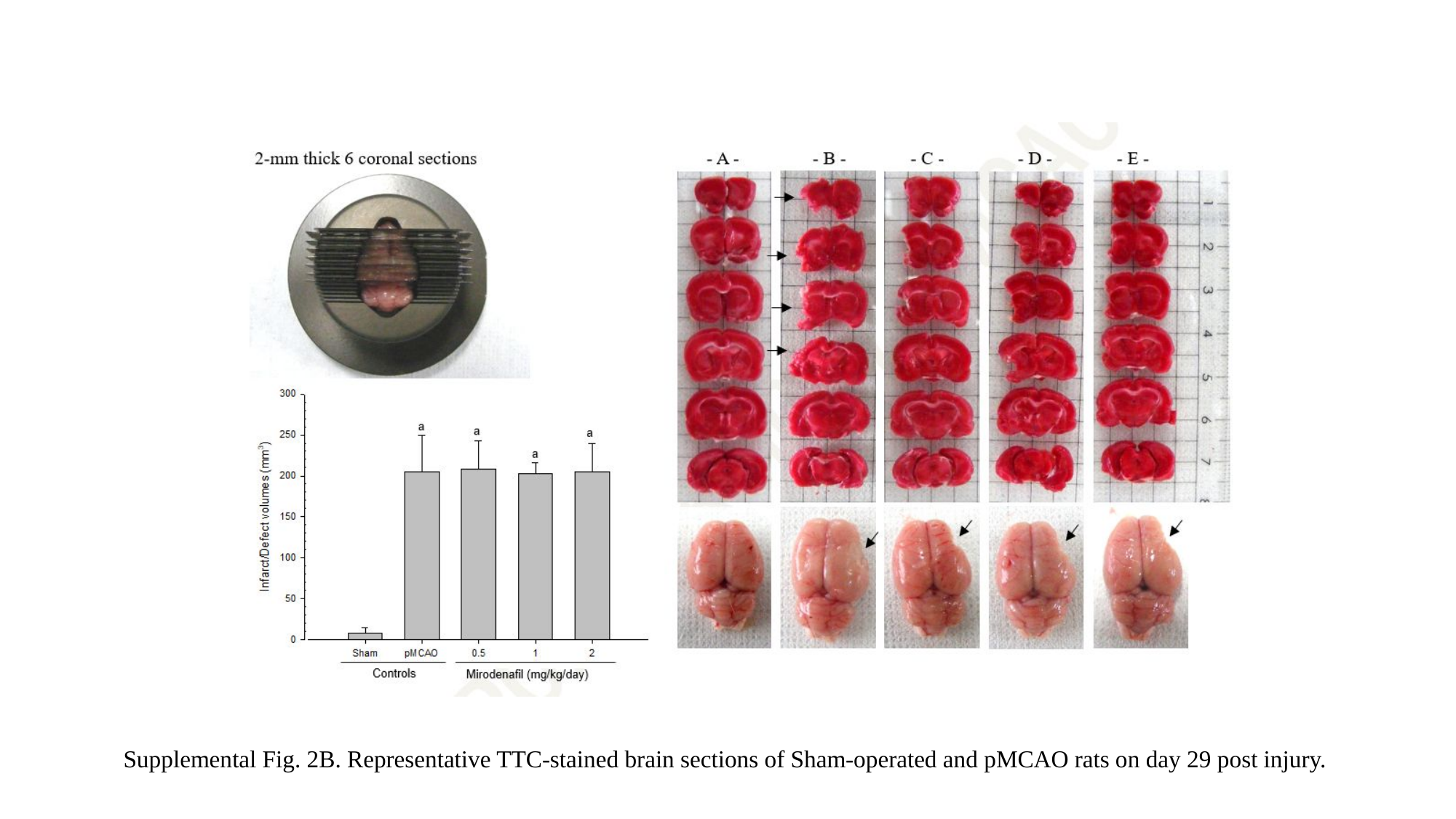

Supplemental Fig. 2B. Representative TTC-stained brain sections of Sham-operated and pMCAO rats on day 29 post injury.
